# Recombinant Myxoma Virus Expressing Walleye Dermal Sarcoma Virus orfC Is Attenuated in Rabbits

**DOI:** 10.3390/v12050517

**Published:** 2020-05-08

**Authors:** Laura V. Ashton, Sandra L. Quackenbush, Jake Castle, Garin Wilson, Jasmine McCoy, Mariah Jordan, Amy L. MacNeill

**Affiliations:** 1 Department of Microbiology, Immunology, and Pathology, Colorado State University, Fort Collins, CO 80523, USA; Laura.Ashton@colostate.edu (L.V.A.); Sandra.Quackenbush@colostate.edu (S.L.Q.); wilsong@rams.colostate.edu (G.W.); Jasmine.McCoy@colostate.edu (J.M.); Mariah.Jordan@colostate.edu (M.J.); 2 Department of Clinical Sciences, Colorado State University, Fort Collins, CO 80523, USA; Jake.Castle@colostate.edu

**Keywords:** poxvirus, myxoma virus, attenuated, rabbits, oncolytic

## Abstract

The poxvirus, myxoma virus (MYXV) has shown efficacy as an oncolytic virus (OV) in some cancer models. However, MYXV replication within murine cancer models and spontaneous canine sarcomas is short-lived. In mice, successful treatment of tumors requires frequent injections with MYXV. We hypothesize that treatment of cancer with a recombinant MYXV that promotes apoptosis could improve the efficacy of MYXV. The orfC gene of walleye dermal sarcoma virus (WDSV), which induces apoptosis, was recombined into the MYXV genome (MYXVorfC). A marked increase in apoptosis was observed in cells infected with MYXVorfC. To ensure that expression of WDSV orfC by MYXV does not potentiate the pathogenesis of MYXV, we evaluated the effects of MYXVorfC inoculation in the only known host of MYXV, New Zealand white rabbits. Virus dissemination in rabbit tissues was similar for MYXVorfC and MYXV. Virus titers recovered from tissues were lower in MYXVorfC-infected rabbits as compared to MYXV-infected rabbits. Importantly, rabbits infected with MYXVorfC had a delayed onset of clinical signs and a longer median survival time than rabbits infected with MYXV. This study indicates that MYXVorfC is attenuated and suggests that MYXVorfC will be safe to use as an OV therapy in future studies.

## 1. Introduction

There is a growing interest in using viruses to eliminate cancers. The first oncolytic virus (OV) approved for use in the United States is marketed for treatment of melanoma in humans [1]. Although there are ongoing clinical trials testing the efficacy of oncolytic virotherapy in many types of human cancers, new OVs that are safer and more globally effective are actively being researched.

The poxvirus, MYXV is an excellent candidate oncolytic virotherapeutic because, unlike other OVs, it does not cause disease in humans or other vertebrates, with the exception of rabbits [2,3,4,5,6,7,8,9]. In spite of its species specificity, MYXV productively infects cultured cancer cells from several animal species [10,11,12,13]. In culture, data suggest that MYXV can replicate in neoplastic cells which have activated Akt [11,14] and lack appropriate Type I interferon responses to virus infection [12,15]. In rodent cancer models, MYXV treatment has eliminated some glioma xenografts [16] and reduced tumor burden of different allografts [17,18,19,20], but to date allografts have not been eliminated by MYXV treatment alone. We hypothesize that a recombinant MYXV with an enhanced oncolytic effect in cell culture will be a more potent anti-cancer therapy. However, before a new MYXV OV is tested in cancer models, its safety profile must be determined in its natural host, rabbits.

This study compares the pathogenesis of wild-type MYXV to a recombinant MYXVorfC virus in New Zealand white rabbits. MYXVorfC expresses walleye dermal sarcoma virus (WDSV) OrfC, which encodes a pro-apoptotic protein. In cell culture, the orfC protein disrupts mitochondrial function and cells exhibit characteristics of apoptosis [21]. It is possible that orfC expression contributes to the tumor regression observed during clinical resolution of WDSV infection. It also is likely that the immune response to WDSV in infected fish plays a critical role in tumor regression [21,22]. Our data indicate that MYXVorfC replicates and enhances apoptosis in permissive cell cultures. In rabbits, clinical signs of virus infection are delayed and median survival time is increased in animals inoculated with MYXVorfC as compared to MYXV. One of six rabbits survived infection with MYXVorfC, while all six rabbits infected with MYXV succumbed to disease. These data indicate MYXVorfC is pro-apoptotic in cell culture and is attenuated in rabbits. These properties support using MYXVorfC in animal models of cancer to determine if MYXVorfC is a more effective OV than wild-type MYXV.

## 2. Materials and Methods

### 2.1. Cell Culture

Rabbit-kidney epithelial (RK-13, ATCC CCL-37) cells originated in Dr. Richard Moyer’s laboratory. The cancer cells evaluated (Morris soft tissue sarcoma (STS) and King osteosarcoma (OSA)) were isolated from canine cancer patient tumors that were surgically removed at the University of Illinois and Colorado State University Veterinary Teaching Hospitals, respectively. Clients from both institutions gave written permission for cells to be isolated. Cells were validated as canine origin by short tandem repeat analysis [23]. Cell growth media was comprised of Minimal Essential Medium with Earle’s salts and 2 mM L-glutamine supplemented with 2 mM L-glutamine, 50 U/mL penicillin, and 50 µg/mL streptomycin (GE Healthcare, Marlborough, MA, USA); 0.1 mM nonessential amino acids and 1 mM sodium pyruvate (Corning, Corning, NY, USA); and 10% fetal bovine serum (FBS; VWR Life Science Seradigm, Radnor, PA, USA). Cells were maintained in a water-jacketed incubator at 5% CO_2_ and 37 °C.

### 2.2. Viruses

**Figure 1:**
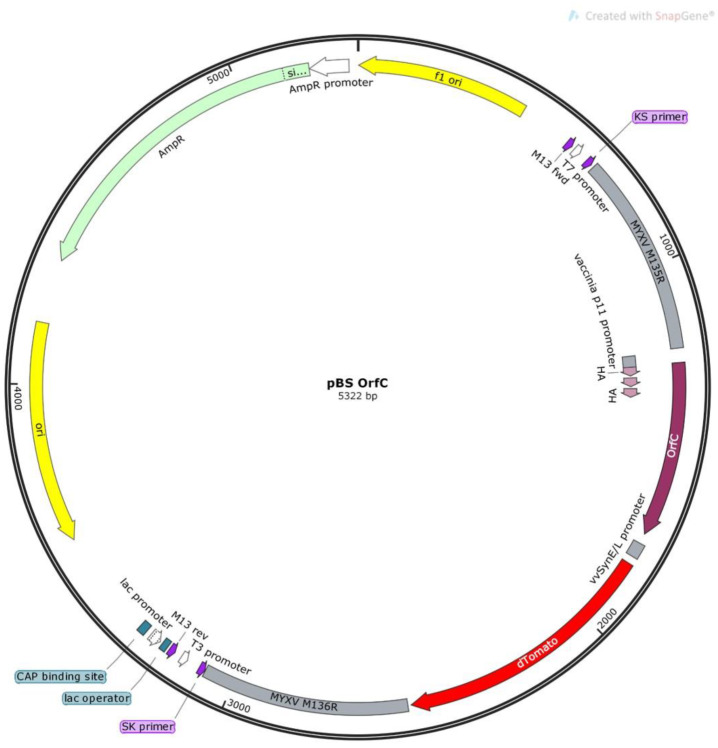
Plasmid design. Diagram (SnapGene, GSL Biotech, San Diego, CA, USA) of the modified pBluescipt plasmid containing the WDSV orfC gene and the tdTomato reporter gene flanked by MYXV M135R and MYXV M136R.

Wild-type MYXV (Lausanne strain) and recombinant MYXV-red (originally designated vMyx-tdTr [24]) was originally acquired from Dr. Grant McFadden. MYXVorfC was constructed by recombining a 2385 base pair (bp) PCR product into wild-type MYXV between the genetic open reading frames M135 and M136 using a pBluescript plasmid vector (Figure 1). Plasmid constructs were amplified by transformation into *Escherichia coli* DH5α chemically competent cells (Rapid5-a, Hardy Diagnostics, Santa Monica, CA, USA). Successful construction was confirmed using restriction enzyme digests and Sanger sequencing. DNA for transfection was prepared using PCR OneTaq Mastermix (New England Biolabs, Ipswitch, MA, USA) containing 0.5 µM of M13 forward primer (5′ GTA AAA CGA CGG CCA GT 3′), 0.5 µM of M13 reverse primer (5′ CAG GAA ACA GCT ATG ACC 3′), and 1.0 µg plasmid DNA. Amplification was performed in a thermocycler under the conditions: 94 °C for 1 min, followed by 30 cycles of 94 °C for 30 sec, 52 °C for 1 min, and 68 °C for 5 min, and a final 68 °C for 5 min step. The PCR product contained DNA sequences of: (1) the 5′ fragment of MYXV M135, (2) tandem dimer tomato red (tdTomato) under the transcriptional control of a synthetic early/late poxvirus promoter (vvSynE/L), (3) hemagglutinin (HA)-tagged WDSV orfC under the control of a late poxvirus promoter (p11), and (4) the 3′ fragment of MYXV M136. Transfection of the PCR fragment was performed using a modified method described by Rice et al. 2011 [25]. Briefly, RK-13 cells were infected at a multiplicity of infection (moi) of 0.01 with wild-type MYXV, transfected with 0.2 µg of PCR product DNA, and combined with Lipofectamine 2000 (Invitrogen, Carlsbad, CA, USA) to potentiate recombination of the PCR product and viral DNA. Cells were scraped into growth media at 72 h post-inoculation (hpi), centrifuged at 400× *g* for 15 min, washed in phosphate buffered saline (PBS), re-suspended in media lacking FBS, frozen and thawed 3 times, and sonicated. Viral lysates were serially diluted in media lacking FBS and incubated on RK-13 cells for 30 min. A solid overlay of 1 part 2× growth media and 1 part 1% agarose was placed on the infected cells. Viral foci that formed were screened for fluorescent red protein expression using a 560/40 nm bandpass excitation filter and a Leica DMI4000B inverted microscope. Fluorescent foci were isolated and expanded in RK-13 cells. The process of picking foci and growing viruses was repeated 9 times until only foci that expressed red fluorescent protein were observed. Viral purification was confirmed using PCR and next generation sequencing. For injection into rabbits, viruses were grown and titered in RK-13 cell cultures. Cellular debris was removed by sucrose pad purification as previously described [26].

### 2.3. Viral Growth Curves

Growth media was removed from wells of RK-13 cells when they were 80% confluent. Cells were inoculated with MYXV or MYXVorfC in media lacking FBS (moi = 0.1, *n* = 10 per group). Cells were incubated with virus for 1 h at 5% CO_2_ and 37 °C. Viral inoculum was removed, cells were rinsed with PBS, and growth media was added to wells. Cells were scraped into growth media at designated time-points post-inoculation, frozen and thawed 3 times, and sonicated. Plaque assays were then performed to determine the number of infectious virions per mL of media. To perform plaque assays, viral lysates were serially diluted in media lacking FBS and incubated on RK-13 cells for 30 min. A solid overlay of 1 part 2× growth media and 1 part 1% agarose was placed on the infected cells. Viral foci were counted 4 days later. The log of plaque/focus-forming units (pfu) per mL was calculated and plotted versus time.

### 2.4. Detection of Exogenous Protein Production by MYXVorfC

Fluorescence from tdTomato protein expression by MYXVorfC was detected using a 560/40 nm bandpass excitation filter and a Leica DMI4000B inverted microscope. Production of the HA-tagged OrfC protein was detected using a Western immunoblot. Briefly, RK-13 cells were grown in 35 mm diameter plates to 90% confluency and inoculated with MYXVorfC (moi = 0.5). Infected cells were collected into cell lysis buffer at several time points pi. Total protein concentration was determined with a standard Bradford assay and 20 μg of protein from each cell lysate was separated using SDS-PAGE (10%). The SDS-PAGE-separated proteins were transferred to a nitrocellulose membrane. Membranes were incubated in blocking buffer for 1 h. Membranes were washed then incubated with rat anti-HA IgG (Milipore Sigma, St Louis, MO, USA) diluted 1:1000 in blocking buffer overnight at 4 °C. Membranes were washed then incubated for 1 h at room temperature with a horseradish peroxidase-conjugated goat anti-rat IgG antibody (ImmunoReagents, Raleigh, NC, USA) diluted 1:1000 in blocking buffer. A chemiluminescent Western immunoblot detection kit (GE Healthcare, Marlborough, MA, USA) was used to detect antigen-antibody complexes. These experiments were repeated a minimum of three times.

### 2.5. Apoptosis in Cell Culture

Growth media was removed from wells of RK-13 cells and cancer cells when they were 80% confluent. Cells were inoculated in media lacking FBS without virus (mock), with MYXV, or with MYXVorfC (moi = 1, *n* ≥ 22 per group). Cells were incubated with virus for 1 h at 5% CO_2_ and 37 °C then growth media was added to the wells. A RealTime-Glo Annexin V Apoptosis and Necrosis Assay kit (Promega, Madison, WI, USA) was used per the manufacturer’s instructions to label cells expressing phosphatidylserine. Luminescence and fluorescence (485 nm excitation/525 nm emission) were detected using a BioTek Synergy H1 microplate reader. The percent luminescent signal from cells undergoing apoptosis and the percent fluorescent signal from partially ruptured (necrotic) cells were graphed relative to mock-infected controls. The ratio of percent apoptotic to percent necrotic cells was also calculated and graphed. 

### 2.6. Rabbits

All animal procedures were approved by the Institutional Animal Care and Use Committee at Colorado State University (protocol #17-7708A). Twelve 8-week-old, female, New Zealand white rabbits were purchased from Western Oregon Rabbit Company (Philomath, OR, USA) and were acclimated for 5 days. Prior to starting the study, all rabbits were deemed healthy by physical examination including determination of heart rate and respiratory rate; auscultation of the heart and lungs; palpation of the abdomen and lymph nodes; measurement of weight and rectal temperature; evaluation of the injection site, haired skin, and mucous membranes for evidence of lesion formation; and assessment of food and water intake, grooming behavior, and mentation. An area of fur on the lateral aspect of the upper right thigh was shaved and disinfected with an accelerated hydrogen peroxide solution. Rabbits were inoculated with virus via an intradermal injection of 50 pfu of sucrose pad purified wild-type MYXV (*n* = 6) or MYXVorfC (*n* = 6) in 100 µL PBS. The area was cleaned with an accelerated hydrogen peroxide solution after the virus is administered. Rabbits were given physical examinations daily. The lesion length, width, and height at the site of inoculation were measured daily. Lesion volume was calculated as volume = length × width × height. The clinical scoring system outlined in Rice et al., JVI 2014 was used [27]. Animals were euthanized as soon as signs of respiratory distress were observed; if rectal temperature was less than 36 °C; if the rabbit appeared mentally dull with poor response to noise or light; if the rabbit stopped grooming, eating, or drinking; or at Day 21 post-inoculation.

### 2.7. Sample Collection

Prior to euthanasia, rabbits were anesthetized with a freshly-mixed combination of 50 mg/kg ketamine and 10 mg/kg xylazine by intramuscular injection into the proximal and lateral aspect of the left thigh. Fully anesthetized rabbits (unresponsive to noxious stimulation, such as pinching the toes with forceps) were administered an overdose of intracardiac pentobarbital (120 mg/kg) to ensure they were deceased. After euthanasia, a necropsy was performed to collect skin lesions and all internal organs. Two sections of each tissue were collected; one was flash frozen in liquid nitrogen for isolation of virus and detection of viral DNA and one was preserved in 10% buffered formalin for histopathology.

### 2.8. Detection of Virus in Tissues

Flash frozen tissue sections were weighed and homogenized. Samples were serially diluted in media without FBS, sonicated, and inoculated onto RK-13 cells for 30 min. A solid overlay of 1 part 2× growth media and 1 part 1% agarose was placed on the infected cells. Viral foci were counted 4–6 days later. Pfu per mg of tissue calculated. Additionally, DNA was isolated from sample homogenates using a tissue DNA isolation kit (DNeasy Blood and Tissue Kit, QIAGEN, Hilden, Germany). Next, 5 µL of DNA was used in PCR reactions with two primer sets. A set of primers that detect a multigenic region of the MYXV genome that includes M032R, M033R, M034L (DNA polymerase), and M035R was used to detect MYXV in tissues (Forward 5′CAC CCT CTT TAG TAA AGT ATA CAC C 3′, Reverse 5′GAA ATG TTG TCG GAC GGG 3′). An 800 bp product is detected for both MYXV and MYXVorfC with these primers. A second set of primers was also used (Forward 5′ ACA TAC GAC ATC GGA CAG CA 3′, Reverse 5′CGT CGA TCG CTG TGT AAG AA 3′) that covered the region of the gene insert (M135-M136); a 320 bp product is expected for wild-type MYXV and a 1547 bp product is expected for MYXVorfC. Amplification was performed in a thermocycler under the conditions: 94 °C for 1 min, followed by 30 cycles of 94 °C for 30 sec, 55 °C for 1 min, 72 °C for 2 min and a final 72 °C for 10 min elongation step.

### 2.9. Histopathology

Formalin-fixed tissue was paraffin embedded, sectioned, and stained with hematoxylin and eosin (H&E) for histologic evaluation. The extent of inflammation, necrosis, and edema observed in skin lesions histologically was graded as previously described [20]. Similarly, additional sections were used to detect apoptosis using a TUNEL (TdT-mediated dUTP Nick End Labeling) assay (Click-iT TUNEL, Life Technologies, Carlsbad, CA, USA) and the extent of apoptosis was graded.

### 2.10. Statistical Analysis

Median survival time was calculated using Kaplan–Meier survival curves. The apoptosis to necrosis ratio in RK-13 cells; clinical scores, respiratory rate, heart rate, body temperature, and lesion volume in rabbits; virus titers in tissues; and the extent of inflammation, necrosis, edema, and apoptosis in histologic sections were compared using t-tests. All data were analyzed using GraphPad Prism version 8.1.0 software (GraphPad, San Diego, CA, USA).

## 3. Results

### 3.1. Validation of MYXVorfC Construction

**Figure 2:**
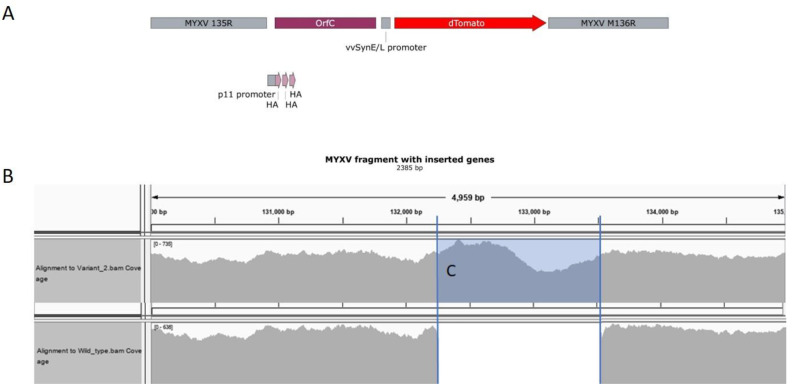
Design and sequence analysis of MYXVorfC. (**A**) Diagram (SnapGene, GSL Biotech, San Diego, CA, USA) of the PCR product that was transfected into MYXV-infected RK-13 cells. (**B**) Alignment of the sequence maps from 130,000–135,000 bp in purified recombinant MYXVorfC DNA as compared to MYXV (AF170726.2) using next generation sequencing. The number of times each nucleotide was read by the sequencer is represented in gray. (**C**) The expected PCR insert was successfully recombined into MYXV to create MYXVorfC. Wild-type MYXV sequence was not detected in the purified MYXVorfC virus sample.

A diagram of the PCR product introduced between genes M135 and M136 to create the recombinant MYXVorfC virus is shown in Figure 2A. Restriction enzyme digestions of MYXVorfC indicated the predicted DNA fragment lengths. MYXVorfC sequence analysis indicated that no unexpected genetic changes were caused during recombination of the PCR fragment into wild-type MYXV (Figure 2B).

**Figure 3:**
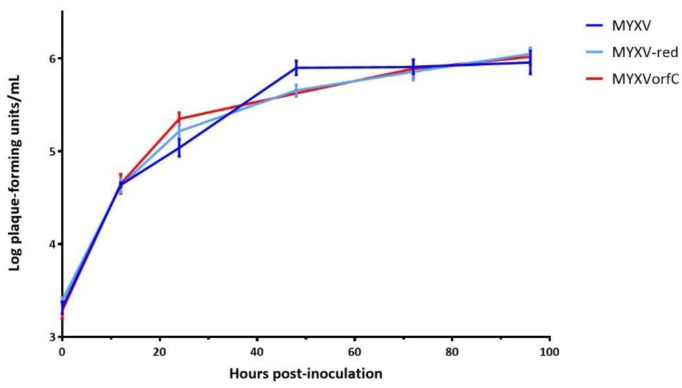
Virus growth curves. Data points represent the average of ten replicates. No significant differences were observed in the growth of wild-type MYXV, MYXV-red, or MYXVorfC (moi = 0.1, error bars = SEM).

**Figure 4:**
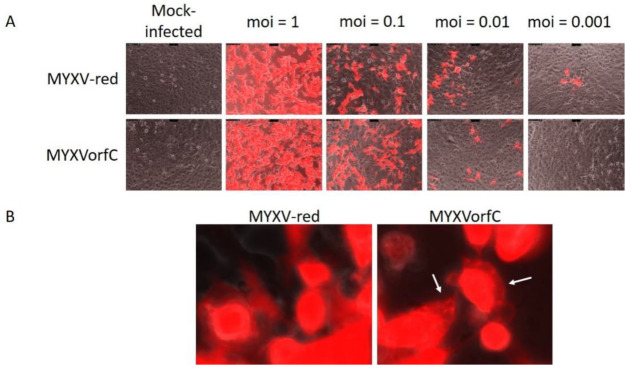
Expression of tdTomato by recombinant MYXV (moi = multiplicity of infection). (**A**) Representative images of infected RK-13 cells 24 hpi (100× magnification). No significant differences in the number of infected cells were observed. (**B**) Morphologic differences in viral plaques grown on RK-13 cells (moi = 0.01, 48 hpi). Some MYXVorfC-infected cells contained large cytoplasmic vacuoles (arrows) which were not detected in cells infected with MYXV-red (800× magnification). Data were repeatable (*n* > 3).

**Figure 5:**
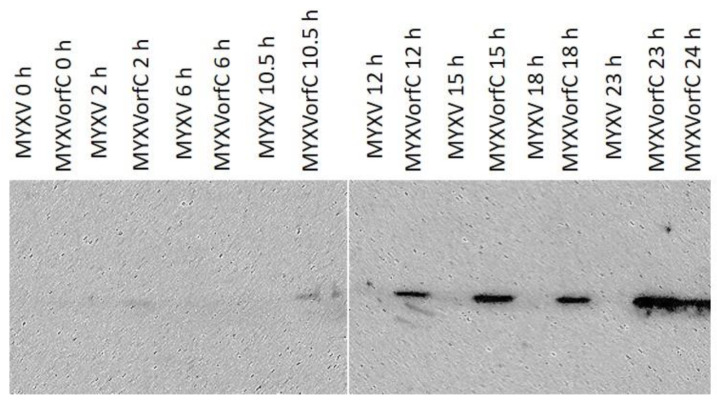
Western immunoblot. Hemagglutinin-tagged orfC was detected in RK-13 cell lysates by 12 hpi (moi = 0.5). Data were repeatable (*n* = 3).

Growth curve kinetics of wild-type MYXV and MYXVorfC in RK-13 cells were statistically indistinguishable (Figure 3), indicating that insertion of the PCR product did not negatively affect MYXVorfC replication in permissive RK-13 cells. Next, protein expression of orfC and tdTomato by MYXVorfC was assessed. Red fluorescence was detected in RK-13 cell cultures inoculated with MYXVorfC (Figure 4). The number of cells expressing tdTomato was similar when MYXV-red and MYXVorfC infections were compared (Figure 4A). However, subtle morphologic differences were noted. Some MYXVorfC-infected cells contained large cytoplasmic vacuoles, which were not observed in MYXV-red-infected cells (Figure 4B). OrfC protein expression was detected by 12 hpi using Western blot analysis (Figure 5). Together, these data indicate that tdTomato and orfC were successfully inserted into wild-type MYXV to create a recombinant MYXVorfC that expresses detectable amounts of tdTomato and orfC proteins.

### 3.2. Apoptosis Is Induced By MYXVorfC

**Figure 6:**
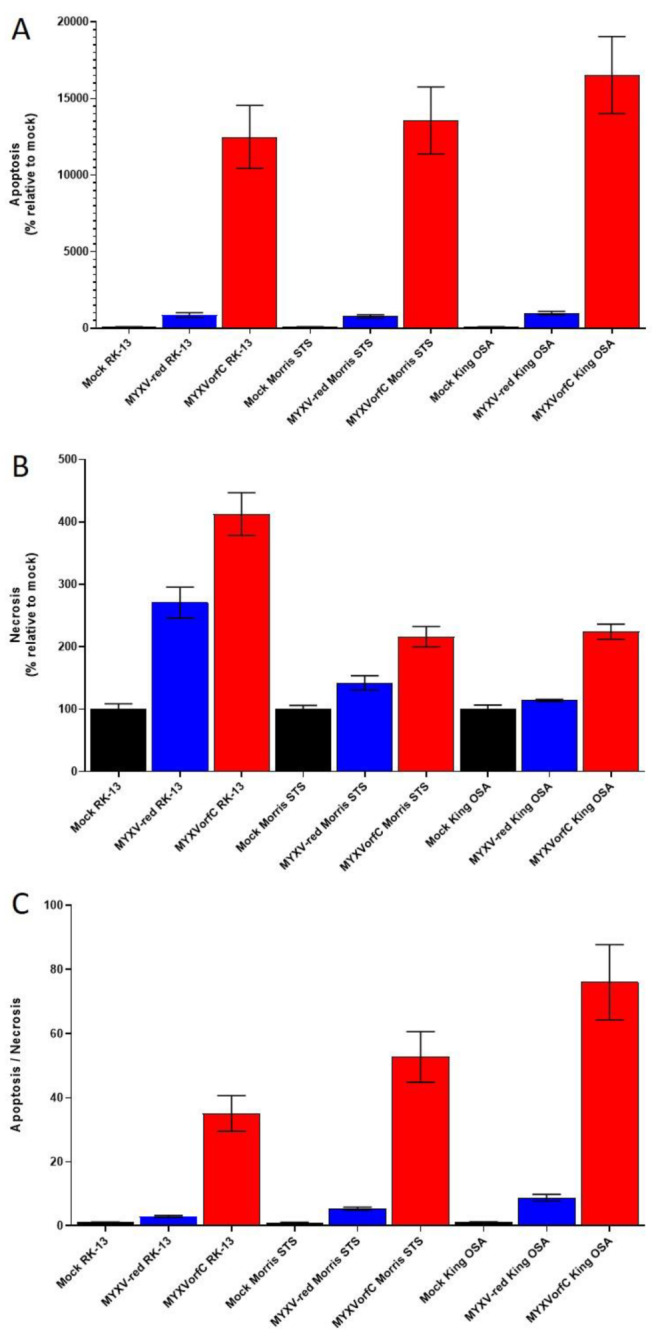
Percent (**A**) apoptosis and (**B**) necrosis relative to mock-infected controls in RK-13 cells and two cancer cells (Morris STS and King OSA). (**C**) The ratio of percent apoptosis and percent necrosis is also shown. Apoptotic and necrotic cells were significantly increased after MYXVorfC infection as compared to mock-controls or MYXV-red infection (*n* ≥ 22, moi = 1, 24 hpi, error bars = SEM, *p*-values ≤ 0.0016)

The purpose of using MYXV to express the orfC protein was to enhance apoptosis in infected cancer cells. Apoptosis was greatly increased in RK-13 cells infected with MYXVorfC as compared to MYXV-red infected or mock-infected cells (Figure 6).

### 3.3. MYXVorfC Is Attenulated in Rabbits

**Figure 7:**
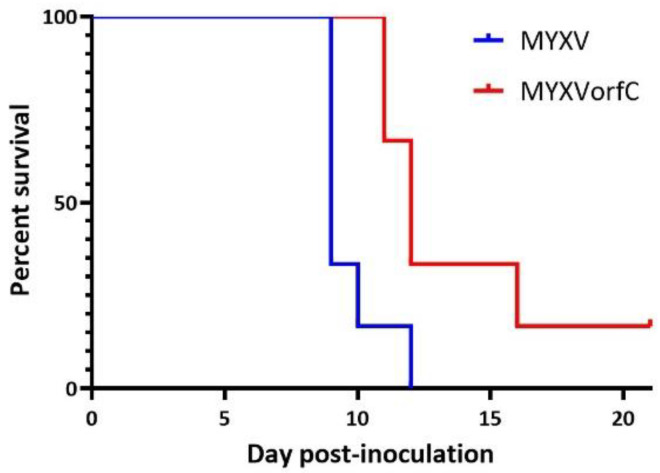
The median survival time of rabbits infected with 50 pfu MYXV was significantly shorter than that of rabbits infected with 50 pfu MYXVorfC ((*n* = 6 per group, *p*-value = 0.014).

As expected, all New Zealand white rabbits infected with 50 pfu wild-type MYXV needed to be euthanized due to respiratory distress. One of six rabbits infected with 50 pfu of MYXVorfC survived. The median survival time of rabbits inoculated with MYXVorfC was significantly greater than that of rabbits inoculated with MYXV (Figure 7). The median survival times for MYXV- and MYXVorfC-infected rabbits were 9 and 12 Days pi (dpi), respectively.

**Figure 8:**
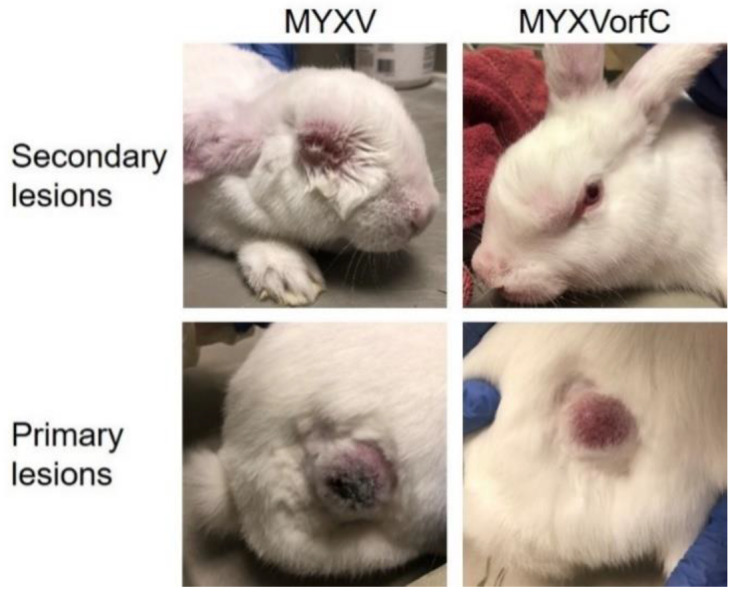
Representative images of gross skin lesions 9 days after intradermal inoculation of 50 pfu MYXV or MYXVorfC on the right thigh

**Figure 9:**
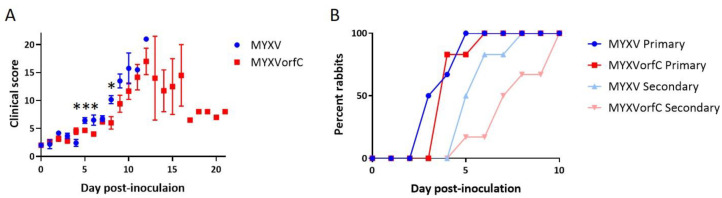
Differences in disease progression in rabbits after intradermal inoculation of 50 pfu MYXV or MYXVorfC (*n* = 6 per group). (**A**) Average daily clinical score (error bars = SEM, * *p*-value < 0.035). (**B**) Day that development of primary and secondary skin lesions was first observed on individual rabbits.

**Figure 10:**
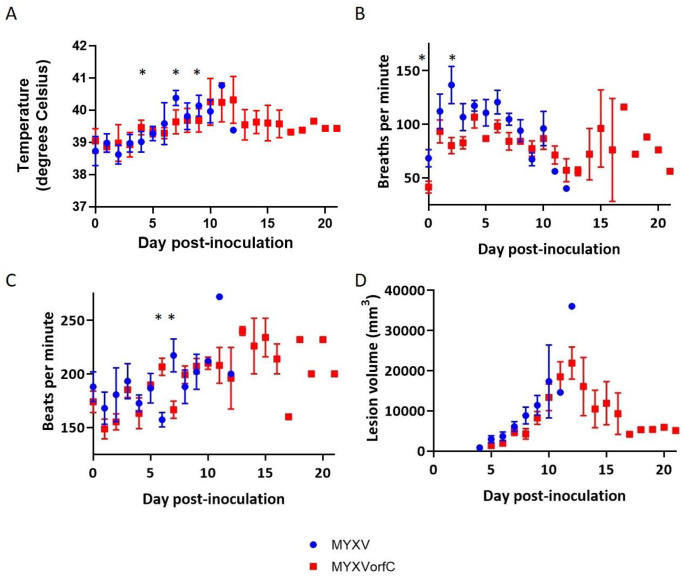
Average daily measurements during physical examination of rabbits infected with 50 pfu of MYXV or MYXVorfC (*n* = 6 per group). Small, but statistically significant differences were observed on a few days when average (**A**) body temperature, (**B**) respiratory rate, and (**C**) heart rate were compared. No significant differences were observed when (**D**) average daily lesion volumes were calculated (error bars = SEM, * *p*-values < 0.046).

Development of clinical signs of disease (including respiratory distress and dissemination of virus to form edematous, ulcerative skin lesions) was slightly delayed in rabbits infected with MYXVorfC (Figure 8). Slightly, but significantly, lower clinical scores were observed in MYXVorfC rabbits on Days 5, 6, and 8 after virus injection (Figure 9A). These differences were attributed to the delay in development of secondary lesions on mucus membranes and ear pinnae in rabbits infected with MYXVorfC (Figure 9B) and detection of fever (body temperature > 40 °C, Figure 10A). Small differences in mean respiratory rate (Figure 10B) and heart rate (Figure 10C) were found on certain dpi, but no definitive trends were seen over time. No significant differences in lesion volume were calculated, but the average volume of the primary lesion of rabbits infected with MYXVorfC decreased in animals that survived beyond Day 12 pi (Figure 10D). These data indicate that MYXVorfC is less pathogenic than wild-type MYXV.

### 3.4. MYXVorfC Tissue Tropism Is Not Altered

**Figure 11:**
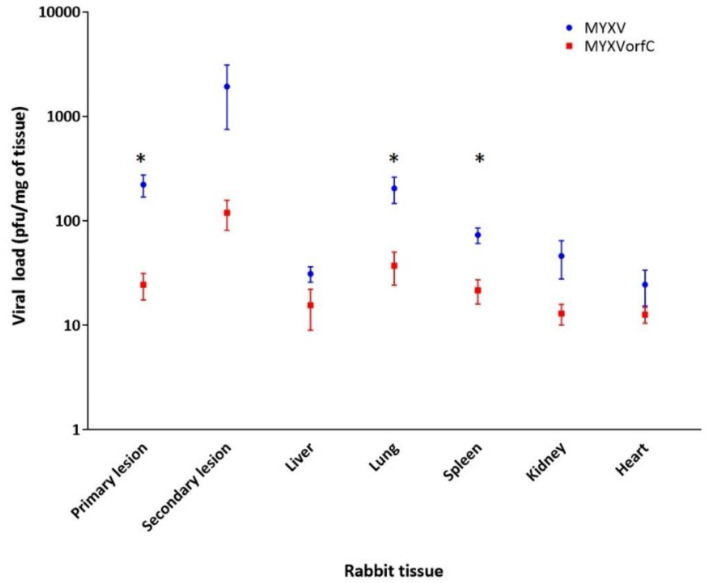
Average virus titer in rabbit tissues. Tissue homogenates from MYXV- and MYXVorfC-infected rabbits at the time of euthanasia contained replicating virus (*n* = 6 per group). Significantly lower titers of MYXVorfC were extracted from primary lesions, lung, and spleen (error bars = SEM, * *p*-values < 0.05)

Dissemination of wild-type MYXV and MYXVorfC during infection of rabbits appeared similar. Viral DNA was detected in skin, lungs, heart, spleen, liver, and kidney in all MYXV-infected rabbits and most MYXVorfC-infected rabbits (Table 1). Viral titers (indicating the presence of infectious virions) were significantly lower in the primary lesions, lungs, and spleen of the MYXVorfC-infected rabbits when compared to MYXV-infected rabbits (Figure 11). Viral DNA could be detected in tissues with titers as low as 2.5 pfu/mg. All tissues with titers > 35 pfu/mg were positive for viral DNA by PCR using both primer sets.

### 3.5. MYXV and MYXVorfC Induce Similar Histologic Lesions

**Figure 12:**
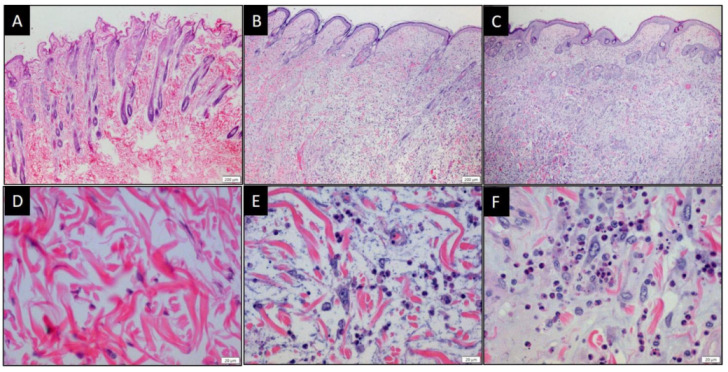
Photomicrographs of skin from the lateral thigh of rabbits injected with (**A**,**D**) saline [28], (**B**,**E**) 50 pfu MYXV, or (**C**,**F**) 50 pfu MYXVorfC. Samples were collected at the time of euthanasia, processed, and stained with H&E (A, B, and C magnification 10× objective; D, E, and F magnification 40× objective).

**Figure 13:**
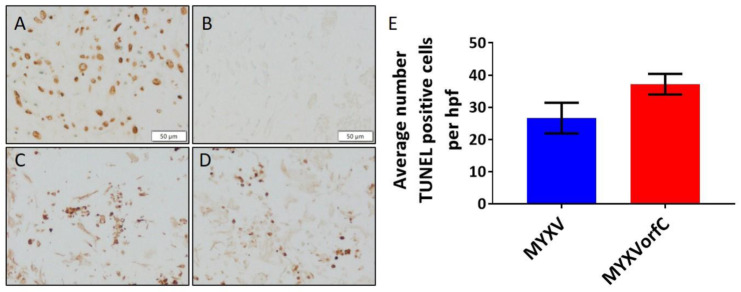
TUNEL staining in primary skin lesions. (**A**) Positive (brown nuclei) and (**B**) negative control samples were adequate. Positive TUNEL staining was observed in both (**C**) MYXV-infected and (**D**) MYXVorfC-infected rabbits (magnification 20× objective; *n* = 6 per group). (**E**) The average number of TUNEL positive cells in ten 40× objective high-power fields (hpf) was calculated. The mean difference between TUNEL staining in primary lesions of MYXV- and MYXVorfC-infected rabbits was not statistically significant (error bars = SEM)

No overt histologic abnormalities were appreciated in heart or kidney sections. Viral pneumonia was observed in lung tissue from all rabbits with mild to moderate edema and diffuse suppurative inflammation. Sloughing of bronchial epithelial cells into the bronchial lumen was noted. Mild hyperplasia of perivascular lymphoid tissue of the lungs was observed in both MYXV- and MYXVorfC-infected rabbits. Evidence of disease in the liver was more variable. Mild diffuse lymphocytic inflammation was observed in the livers of 67% (4/6) MYXV-infected rabbits and 33% (2/6) MYXVorfC-infected rabbits. Mild diffuse mixed inflammation (including neutrophils, histiocytes, lymphocytes, and plasma cells) was observed in the liver of 50% (3/6) MYXVorfC-infected rabbits. No inflammation was observed in the liver of the remaining 33% (2/6) MYXV-infected and 17% (1/6) MYXVorfC-infected rabbits. Tissue sections from primary and secondary skin lesions of all rabbits had the classic appearance of myxomatosis in European rabbits. A large focus of full-thickness dermal necrosis was associated with several large reactive fibroblasts and myxedema (Figure 12). Fibroblasts and epithelial cells sometimes contained a large basophilic cytoplasmic viral inclusion. Marked suppurative inflammation was present within the necrotic lesion and moderate mononuclear inflammation surrounded the damaged tissue. The average number of TUNEL positive cells in ten 40× objective microscopic fields in sections of primary skin lesions was not significantly different in MYXV- and MYXVorfC-infected rabbits (Figure 13). The secondary lesion from one MYXVorfC-infected rabbit was beginning to heal with extension of the epidermis under a layer of fibrin and cellular debris.

### 3.6. MYXVorfC Stimulated Follicular Hyperplasia in Lymphoid Tissues

**Figure 14:**
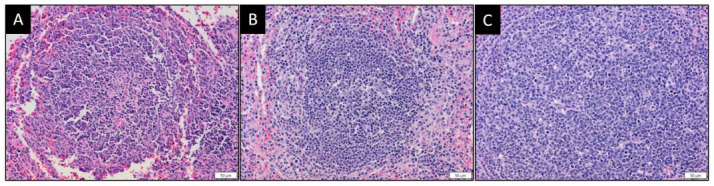
Representative photomicrographs of splenic lymphoid follicles from rabbits injected with (**A**) saline, (**B**) 50 pfu MYXV, or (**C**) 50 pfu MYXVorfC. Samples were collected at the time of euthanasia. Marked follicular hyperplasia was noted in rabbits infected with MYXVorfC (H&E, magnification 20× objective).

Spleen and lymph nodes from uninfected rabbits and rabbits infected with MYXV had minimal lymphoid reactivity (Figure 14A,B). In contrast, MYXVorfC-infected rabbits developed lymphoid hyperplasia (Figure 14C) suggestive of a more activated adaptive systemic immune response to the virus.

## 4. Discussion

Development of safe, effective OVs could revolutionize cancer treatment, particularly for neoplasms that are refractory to currently available therapies. MYXV is nonpathogenic in humans [6] and shows potential as a safe OV [29,30]. However, the effectiveness of MYXV is somewhat limited. We predict that OVs that induce apoptosis of tumor cells quickly can expose the immune response to tumor antigens more rapidly and negate limitations of rapid removal of MYXV (which we hypothesize is due to the anti-viral response induced by healthy cells within the tumor microenvironment). With this in mind, we designed a pro-apoptotic, recombinant MYXV, MYXVorfC.

MYXVorfC contains an HA-tagged orfC gene isolated from WDSV. WDSV orfC may promote seasonal tumor regression in fish infected with WDSV by inducing cytochrome C release from mitochondria [21]. Expression of orfC by MYXVorfC was placed under the control of a late poxvirus promoter element (vaccinia p11) with the expectation that complete replication of MYXVorfC and production of infectious virions would occur before significant apoptosis was induced in infected cells. Additionally we engineered MYXVorfC to encode the reporter element tdTomato under the control of an early/late poxvirus promoter (vvSynE/L) to enable us to screen for and plaque purify recombinant virus. Our sequencing data indicated that the HA-tagged orfC gene and the reporter element tdTomato were successfully recombined into wild-type MYXV. The virus growth curve in RK-13 cells was not altered by recombination of tdTomato or HA-orfC into MYXV. Protein expression of tdTomato was observed as early as 8 hpi and HA-tagged orfC was detected by 12 hpi. Importantly, MYXVorfC significantly increased apoptosis in cell culture as compared to wild-type MYXV. These data indicate that MYXVorfC is able to replicate in cells, express exogenous proteins, induce apoptosis, spread to neighboring cells, and continue to replicate until all cells have been infected. Apoptosis did not reduce the virus growth rate because virus particles that are formed during infection remain infectious when the cells loose viability.

Next, we infected New Zealand white rabbits with MYXVorfC to ensure that the pathogenicity of the recombinant virus was not more severe than wild-type MYXV. During wild-type MYXV infection, virus replicates to high titers at the primary site of virus inoculation. Within 2 to 4 dpi, viremia occurs allowing for systemic dissemination of infectious viral particles. MYXV localizes to the skin and mucous membranes by 5 dpi, replicates to high titers, and causes secondary myxomatous lesions, fever, and viral pneumonia. This leads to severe obstruction of the respiratory tract, respiratory arrest, and death between 8 and 12 dpi [28]. In comparison, rabbits infected with MYXVorfC had a significantly longer median survival time. Five animals required euthanasia between 11 and 16 dpi, but one animal recovered from MYXVorfC infection. Differences in overall clinical scores were minimal and mostly due to a short delay in the development of secondary lesions and fever in rabbits infected with MYXVorfC. The clinical similarities in infected rabbits are not unexpected, as no MYXV genes were disrupted by insertion of orfC into the virus. Therefore, any attenuation observed was due to expression of orfC by MYXVorfC.

Although MYXVorfC infection was attenuated, the dissemination of MYXVorfC was similar to MYXV. Virus DNA and infectious virions were detected for both viruses in all organs tested. However, significantly lower MYXVorfC titers were recovered from the primary skin lesion at the site of inoculation, the lung tissue, and the spleen. This could reflect a mild reduction of MYXVorfC replication in rabbit tissues or increased clearance of the virus by the immune response. MYXVorfC rabbits had clear evidence of lymphoid hyperplasia, which was lacking in MYXV-infected rabbits. This suggests that rabbits mount a more effective immune response against MYXVorfC. Lymphocytic inflammation is also observed during regression of tumors caused by WDSV [22]. If lymphocytic inflammation is induced by orfC expression during oncolytic virotherapy, it could greatly improve outcomes for cancer patients being treated with MYXVorfC.

MYXVorfC was engineered to increase apoptosis during infection. This was confirmed using RK-13 cells in tissue culture. In rabbits, the average number of TUNEL positive cells in MYXV-infected primary lesions (26.7 ± 11.7 /hpf) was lower than that of MYXVorfC-infected lesions (37.2 ± 7.1 /hpf). Although this difference was not statistically significant, a slight increase in TUNEL positive cells was consistently observed in MYXVorfC-infected tissue. It is possible that MYXV-induced apoptosis will be more enhanced in cancerous cells.

In conclusion, MYXVorfC is pro-apoptotic in cell culture and attenuated in rabbits. The marked lymphoid hyperplasia observed in rabbits infected with MYXVorfC suggests that expression of orfC allows rabbits to mount a more effective immune response against MYXVorfC than wild-type MYXV. A very mild increase in apoptosis at the site of MYXVorfC inoculation was also observed. It is possible that the combination of increased immunogenicity and apoptosis contribute to the decreased pathogenicity of the virus. Future studies will test MYXVorfC treatment in animal cancer models to determine if MYXVorfC is more effective than MYXV at eliminating cancer.

## Figures and Tables

**Table 1:** Detection of MYXV or MYXVorfC by PCR in rabbit tissues collected at the time of euthanasia from rabbits inoculated with 50 pfu MYXV or MYXVorfC (*n* = 6 per group).

		Viral DNA Targets of Primers	
Virus	Tissue	Multigene	M135-M136
MYXV	Primary lesion	6/6 (100%)	6/6 (100%)
	Secondary lesion	6/6 (100%)	6/6 (100%)
	Heart	6/6 (100%)	6/6 (100%)
	Kidney	6/6 (100%)	6/6 (100%)
	Liver	6/6 (100%)	6/6 (100%)
	Lung	6/6 (100%)	6/6 (100%)
	Spleen	6/6 (100%)	6/6 (100%)
MYXVorfC	Primary lesion	6/6 (100%)	6/6 (100%)
	Secondary lesion	6/6 (100%)	6/6 (100%)
	Heart	3/6 (50%)	3/6 (50%)
	Kidney	5/6 (83%)	5/6 (83%)
	Liver	6/6 (100%)	5/6 (83%)
	Lung	6/6 (100%)	5/6 (83%)
	Spleen	4/6 (67%)	3/6 (50%)
